# Utility of Artificial Intelligence–Generative Draft Replies to Patient Messages

**DOI:** 10.1001/jamanetworkopen.2024.38573

**Published:** 2024-10-14

**Authors:** Eden English, Janelle Laughlin, Jeffrey Sippel, Matthew DeCamp, Chen-Tan Lin

**Affiliations:** 1 Division of General Internal Medicine, University of Colorado School of Medicine, Denver; 2 University of Colorado Health Medical Group, Longmont; 3 Division of Pulmonary Medicine, University of Colorado School of Medicine, Denver; 4 Center for Bioethics and Humanities, University of Colorado, Denver

## Abstract

This quality improvement study analyzes the usefulness of patient message replies drafted by artificial intelligence for various health care practitioners.

## Introduction

Messages from patients to care teams via patient portals are increasing^1^ and may be associated with burnout.^2^ Health systems are deploying large language models (LLMs) to address this issue by drafting replies to patient messages.^3,4,5^

In contemporary team-based care, how useful are LLM’s to different team members? What iterative changes in an LLM prompt might improve the usefulness of the draft reply?

## Methods

This prospective quality improvement (QI) study was determined to be exempt from review and the requirement of informed consent by the University of Colorado institutional review board. The QI study was conducted from September 2023 to March 2024 within a large academic health system (UCHealth), following the Standards for Quality Improvement Reporting Excellence (SQUIRE) reporting guideline. We deployed an LLM, GPT-4 (Generative Pretrained Transformer 4 [OpenAI]), via the electronic health record (Epic Systems). Internally dubbed PAM Chat (Patient Advice Message Chatbot reply), the LLM drafted a reply to incoming messages from the patient portal in 9 clinics (6 primary care and 3 specialty), for nurses, medical assistants (MAs), and clinicians (physicians and advance practice clinicians [APCs]).

Based on feedback during the project, we iteratively improved prompts (see the eMethods in Supplement 1 for prompt versions and rationale). For example, we included the assessment and plan (A/P) of the last progress note written by the recipient clinician, emphasizing conciseness. We disclosed that content was computer generated before editing by the clinician to patients.

We surveyed all users 2 weeks after all clinics were live with PAM Chat, including the net promoter score (NPS) and unvalidated de novo items assessing perceptions of the tool. Analysis of variance was used to test the null hypothesis that attitudes toward PAM Chat would not vary by professional role. Data analysis was conducted using JMP Pro software version 17.0.0 (SAS Institute) and took place from April to June 2024. The threshold for significance was a 1-sided *P* < .05.

## Results

PAM Chat generated 21 323 drafts, and 2596 drafts were used (12%) across all 166 users (12 nurses, 14 MAs, and 93 clinicians). Nurses had more favorable views of PAM Chat (Table 1). Nurses were more likely to agree that PAM Chat reduced the need to forward messages to physicians and APCs and helped them to stay within their scope of practice. Of all nurses, 11 (92%) believed that PAM Chat helped improve efficiency, empathy, and tone. Nurses were most likely to recommend PAM Chat to others (NPS, 58). MAs, physicians, and APCs were less favorable.

**Table 1.** Comparison of Survey Respondents (Registered Nurses, Medical Assistants, and Clinicians) Who Replied Agree or Strongly Agree

Question	Respondents, No. (%) (N = 69)			*P* value (χ^2^)
	Registered nurses (n = 12)	Medical assistants (n = 14)	Clinicians (n = 43)^a^	
“I can reply more quickly.”	11 (92)	7 (50)	20 (46)	.03 (10.9)^b^
“It’s easier to express written empathy.”	11 (92)	6 (43)	18 (42)	.01 (13.1)^b^
“The message tone is appropriate.”	11 (92)	11 (79)	28 (65)	.21 (5.9)^b^
“PAM Chat is fun to use.”	11 (92)	8 (57)	22 (51)	.08 (8.2)^b^
“Minimal risk of giving incorrect information.”	8 (67)	2 (14)	12 (28)	.04 (10.1)^b^
“Significant edits are rarely needed.”	NA	NA	5 (12)	<.001 (NA)^c^
“I can address more messages without forwarding.”	9 (75)	3 (21)	NA	.002 (12.8)^b^
“This helps me to stay within my scope of practice.”	6 (50)	4 (29)	NA	.01 (9.0)^b^
Net promoter score (likely to recommend to others)	58	−29	−43	.004 (NA)^d^

Abbreviations: NA, not applicable; PAM Chat, patient advice message chatbot.

^a^ Clinicians included physicians and advance practice clinicians.

^b^ χ^2^ Likelihood ratio.

^c^ Two 1-sided *t* test for single-group measurement.

^d^ Analysis of variance.

Comments from colleagues about PAM Chat were mixed: “Sometimes the answers are astoundingly helpful, other times they are not helpful at all.” Including the A/P from the last note made some replies useful. Illustrative comments are in Table 2.

**Table 2.** Example PAM Chat Replieson

Incoming patient message	PAM Chat draft reply^a^	Comments
“Subject: Knuckle swallow in the morning Message: What should i do about this...hope to hear from you soon...thank you.”	“Hello [Name], Swelling in the knuckles in the morning is a common symptom of rheumatoid arthritis. It’s important to continue taking your prescribed medications, as they can help manage this symptom. You can also try some gentle hand exercises and applying a warm compress to your hands in the morning to help reduce the swelling. If the swelling continues or worsens, please make an appointment so we can evaluate your symptoms and adjust your treatment plan if necessary. Take care.”	The incoming patient message was incredibly brief and had a misspelled word (swallow vs swollen). AI was able to elucidate the question and used the diagnosis from the last progress note in the response.
“Subject: Check in Message: Hi Dr, I have a note on my calendar today to update you about menopause symptoms. The hot flashes have really subsided and I’m doing well overall. Hope things are good with you! Thank you.”	“Hello [Name], I’m glad to hear that your menopause symptoms, particularly the hot flashes, have subsided and that you’re doing well overall. It’s important to continue with the plant-based diet and regular exercise as these can help manage any remaining or future symptoms. Take care.”	The last progress note suggested a plant-based diet to manage symptoms.
“Subject: Unusual discharge from nose Message: Hi. Sunday morning I had a spontaneous discharge from my left nostril unlike anything I’ve experienced before. It was a thin yellowish liquid that steadily trickled out like a pretty good bloody nose…I’m scheduled to see you on the 20th for my annual. If there is anything you think I should do before then, let me know. It just seems so unusual. Thanks.”	“Hello [Name], I’m sorry to hear about your unusual nasal discharge. It could be due to a variety of reasons, including a sinus infection or allergies. However, it’s also important to rule out a cerebrospinal fluid leak, which can sometimes present with clear, watery nasal discharge. I recommend you come in for an appointment as soon as possible so we can evaluate your symptoms and determine the best course of action. Take care.”	Although potentially appropriate for a neurosurgery clinic reply, this response was not clinically relevant here. PAM Chat does not have access to clinic specialty.
“Subject: Wegovy Message: That makes sense. I will avoid the compounded medication. I wanted to send you a Harry and David basket for the holidays. What is a good address to send it to? Thanks!!”	“Hello [Name], Thank you for your message. I appreciate your kind gesture, but as a health care provider, I must maintain professional boundaries and cannot accept personal gifts. Your thoughtfulness is more than enough. Take care.”	AI was empathetic and appropriate and gently declined the offered gift.

Abbreviations: AI, artificial intelligence; PAM Chat, patient advice message chatbot.”

^a^ All outgoing PAM Chat drafts include the language: “Automatically generated and then edited by [Clinician or staff name].”

## Discussion

In this QI study, nurses liked PAM Chat more than MAs and physicians or APCs, with more than 90% believing that it improved efficiency, empathy, and tone. Our results diverge from other reports^3,4,5^ that describe primary care nurses’ negative views of LLMs. This finding may be because the specific messages each group sees are different. Physicians and APCs may preferentially receive complex messages, which are more difficult for the LLM; MAs may view messages containing clinical information as out of scope. This role-based difference and our overall use rate of 12% suggests that, moving forward, LLMs may need to be tuned to recognize who will receive the message (MA, nurse, or physician or APC) and create a reply accordingly.

Our findings also suggest that implementing PAM Chat with ethics principles (eg, transparency) and including the last A/P note can improve utility. Interestingly, in response to a message about a gift, the LLM generated a response that suggested that gift-receiving is not permitted—an AI oversimplification given that the American Medical Association Code of Ethics permits gifts in limited circumstances.^6^

This evaluation was conducted in 9 clinics in 1 health system and may not be generalizable to all health systems. We hope sharing our prompts and results will support ongoing improvement of LLMs in the pursuit of high-quality, safe, and effective patient care while reducing care team burden.

## Notes

Supplement 1. **eMethods.** PAM Chat Prompt Changes and Current General Prompt

Supplement 2.
Data Sharing Statement
