# Error in Methods

**DOI:** 10.1001/jamanetworkopen.2024.51866

**Published:** 2024-11-26

In the Research Letter titled “Utility of Artificial Intelligence–Generative Draft Replies to Patient Messages,”^1^ published October 14, 2024, there was an error in the Methods section, which reported that the health system in which the study was conducted was University of Colorado Health. This should be UCHealth. This article has been corrected.^1^

## References

[1] English E, Laughlin J, Sippel J, DeCamp M, Lin CT. Utility of artificial intelligence–generative draft replies to patient messages. JAMA Netw Open. 2024;7(10):e2438573. doi:10.1001/jamanetworkopen.2024.38573 39401041 PMC11581472

