# Supplementary material for: Utility of Artificial Intelligence–Generative Draft Replies to Patient Messages
Source: JAMA Netw Open. 2024 Oct 14;7(10):e2438573. doi: 10.1001/jamanetworkopen.2024.38573 (PMC11581472; doi:10.1001/jamanetworkopen.2024.38573)
Supplement: Supplement 2. — Data Sharing Statement [file jamanetwopen-e2438573-s002.pdf]

Data Sharing Statement

English. Utility of Artificial Intelligence–Generative Draft Replies to Patient Messages. *JAMA Netw Open*. Published October 14, 2024. doi:10.1001/jamanetworkopen.2024.38573

Data
Data available: No
